# Modulatory Effect of Lifestyle-Related, Environmental and Genetic Factors on Paraoxonase-1 Activity: A Review

**DOI:** 10.3390/ijerph20042813

**Published:** 2023-02-05

**Authors:** Dominika Kunachowicz, Milena Ściskalska, Marta Kepinska

**Affiliations:** Department of Pharmaceutical Biochemistry, Division of Biomedical and Environmental Sciences, Faculty of Pharmacy, Wroclaw Medical University, Borowska 211A, 50–556 Wrocław, Poland

**Keywords:** paraoxonase, PON1 activity, antioxidants, enzyme activity regulation, PON1 polymorphisms

## Abstract

Paraoxonase-1 (PON1) is a calcium-dependent, HDL-bound serum hydrolase active toward a wide variety of substrates. PON1 displays three types of activities, among which lactonase, paraoxonase, arylesterase and phosphotriesterase can be distinguished. Not only is this enzyme a major organophosphate compound detoxifier, but it is also an important constituent of the cellular antioxidant system and has anti-inflammatory and antiatherogenic functions. The concentration and activity of PON1 is highly variable among individuals, and these differences can be both of genetic origin and be a subject of epigenetic regulation. Owing to the fact that, in recent decades, the exposure of humans to an increasing number of different xenobiotics has been continuously rising, the issues concerning the role and activity of PON1 shall be reconsidered with particular attention to growing pharmaceuticals intake, dietary habits and environmental awareness. In the following manuscript, the current state of knowledge concerning the influence of certain modifiable and unmodifiable factors, including smoking, alcohol intake, gender, age and genotype variation on PON1 activity, along with pathways through which these could interfere with the enzyme’s protective functions, is presented and discussed. Since exposure to certain xenobiotics plays a key role in PON1 activity, the influence of organophosphates, heavy metals and several pharmaceutical agents is also specified.

## 1. Introduction

The three-gene paraoxonase family is composed of three members: PON1, PON2 and PON3, which share about 70% genetic sequence homology and 60% identical amino acid sequence [1]. In humans, genes encoding PONs are located on the long arm of chromosome 7, in position 7q21.3–22.1. PONs—in particular, PON1—are hydrolases characterized by one of the broadest known substrate ranges and display various types of activities, among which lactonase (LACase), including thiolactonase and paraoxonase (POase), also referred to as phosphotriesterase and arylesterase (AREase), can be distinguished. The name paraoxonase derives from the ability of these enzymes to hydrolyze paraoxon—highly toxic parathion’s active metabolite—although more recent research indicates that PON1 is not particularly efficient toward this substrate in vivo. PON1 and PON3 are plasma enzymes, while PON2 is intracellular. PON1 is most studied, whereas PON2 seems to be the oldest family member from which PON3 and PON1 have evolved [2,3]. Initially, PON1 belonged to A-esterases due to its capability of catalytic hydrolysis of organophosphate (OP) substrates according to Aldridge’s division [4], and currently is classified as an aryldialkylphosphatase (EC 3.1.8.1) by the Enzyme Commission of the International Union of Biochemistry and Molecular Biology [5].

Owing to multiple activities exerted by PON1 on different substrates, its multifaceted function in humans is not to be underestimated. Not only is PON1 the major detoxifying factor in encounters with OPs or heavy metals, which was its first reported function, but it is also a part of the antioxidant system and displays anti-inflammatory and antiatherogenic properties. It is now known that PON1 serum concentration and activity are highly variable among individuals, and both genetic and environmental factors contribute to “PON1 status” [5]. Although the PON1 genotype is estimated to determine its serum levels and activity in about one fourth [6], epigenetic regulation and exposure to certain xenobiotics plays a vital role in PON1 activity mediated by numerous direct and indirect mechanisms.

A large number of studies has been devoted to exploring the influence of various factors on PON1 activity, a high level of which is vital in providing protection against free radicals or exogenous compounds. Over the past decades, the exposure of humans to an increasing number of different xenobiotics has been continuously rising [7]. Today, the high level of environmental pollution, fast pace of life and excessive stress contributes to development of various diseases, which in turn cause the number of taken pharmaceuticals to grow. All these factors are known to influence PON1 activity. Given that PON1 is essential to maintain oxidative–antioxidative balance and homeostasis [8], the issues concerning its activity shall be reassessed. This manuscript aims to review the current state of knowledge concerning the influence of certain modifiable and unmodifiable factors, including exposure to heavy metals and OPs, diet, smoking, gender, age and genotype variation on PON1 activity, along with pathways through which these could interfere with the enzyme’s protective functions. We will also focus on commonly used pharmaceuticals in relation to diseases, the treatments in which they are used and present results of the most recent in vitro and in vivo human studies. Taking into consideration numerous reports on PON1 complex roles in human homeostasis maintenance, identifying the factors affecting PON1 activity and levels and understanding the manners in which they act seems to be fundamental in elucidating numerous physiological and pathological processes in detail. Additionally, clarification of these mechanisms would be essential for potential therapeutic applications of the enzyme being developed in the future and for efficient disease management. For example, a novel therapeutic strategy based on CRISPR/Cas9 technology in order to reverse atherosclerotic processes through the transcriptional activation of endogenous PON1 and apolipoprotein-A1 (apo-A1) and thereby improve the HDL protective function has recently been evaluated on Caco-2 enterocytes [9]. The study gave promising results and is believed to bear potential for clinical application as the upregulation and subsequent overexpression of PON1 provides protection from oxidative and inflammatory stress and might in the future be an alternative therapy enabling to circumvent often inconvenient side effects of conventional pharmaceuticals used today as a standard. We believe that this review would be helpful in defining the aims and direction of further research concerning PON1.

## 2. PON1 Structure and Physiological Functions

### 2.1. Structure and Function of Human PON1

PON1 is a glycoprotein composed of 355 amino acids with a molecular weight of about 43 kDa [2]. X-ray crystallography enabled the determination of its structure as a six-bladed β-propeller, each containing four β-strands connected by a disulphide bridge, with three surface helices situated at the top of the propeller [10]. The central tunnel contains two calcium ions spaced 7.4 Å apart: one of them (Ca1) is located at the bottom of the enzyme’s active site and exposed to a solvent that is catalytic due to its crucial role in maintaining PON1 enzymatic activity, and the other (Ca2), more tightly bound and buried deeper into the tunnel, is referred to as structural and responsible for the enzyme’s conformational stability. The removal of Ca2 causes PON1 structural destruction, while the reversible elimination of Ca1 results in a loss of the enzyme’s catalytic activity, which is regained after Ca^2+^ ions are added [11]. It has been established that the active site is common for the three PON1 activities (paraoxonase, lactonase and arylesterase) [10,12]. PON1 contains the highly flexible loop comprised of 70–81 amino acid residues, which forms a specific lid closing around the enzyme’s active site while a ligand is bound. The hydrophobic region located below the loop was found to be crucial in PON1 functionality—by enclosing the substrate in such a unique “cage”, the access of solvents is restricted, enabling the hydrolysis of lactones which are hydrophobic and neutral organophosphates. However, molecular details on enzymatic activation induced by interactions between proteins and lipids are still largely unknown and require further studies [13].

Among three cysteine (Cys) residues, Cys42 and Cys353 bound with a disulphide bridge seem to be essential for maintaining PON1 secretion and catalytic activity, which is supported by the evidence that their substitution with alanine led to PON1 inactivation and limited its secretion, whereas Cys284, located near the enzyme’s active site, accounts for PON1 antioxidative properties on low-density lipoproteins (LDL) [14]. Four potential N-glycosylation sites on PON1 are likely to improve its stability, solubility and limit non-specific binding to cell membranes [10]. A histidine dyad (His-115 and His-134) is now suspected to be involved in proper substrate orientation and binding, although it was formerly proposed to be a part of the catalytic mechanism of phosphotriesterase and esterase activities, where a molecule of water was deprotonated and thereby an attacking hydroxyl radical was formed [15,16].

PON1 is synthesized in the liver, and is afterwards secreted into the circulation where it associates with high-density lipoprotein (HDL) particles. In terms of promoting PON1 secretion and stabilizing the released peptide, HDL offers the best physiological acceptor complex [17]. From the structural studies, it is suspected that the N-terminal hydrophobic sequence of the enzyme, which forms a signal peptide retained during maturation except for the initiator methionine residue, has a crucial role in the anchoring of PON1 in the HDL molecule. An amphipathic helix within the active site (H2) is also involved in the binding [18,19]. Ultracentrifugation has shown that PON1 preferentially associates with small dense HDL particles comprising the HDL_3_ subclass, which makes this fraction possess the strongest antioxidant properties [18]. According to the current state of knowledge, HDL–PON1 association is presumably dependent on specific interactions with apo-A1—a major HDL structural component which occurs with a high affinity. Moreover, this interaction results in PON1 stability improvement and enhances its lactonase activity; thereby, apo-A1 was suggested to be the PON1 physiological acceptor [3,20]. A small amount of plasma PON1 is also detected in very low-density lipoproteins (VLDL) and chylomicrons [21,22], and these complexes account for about 5% of the total PON1 activity [3].

The binding of PON1 to HDL is not a permanent feature. PON1 is considered to exist in two forms: HDL-bound and free, which remain in equilibrium, and it was demonstrated that the activity of free PON1 is noticeably lower in comparison to the HDL-associated [12,18]. Interestingly, it was discovered that HDL-associated PON1 is able to redistribute from HDL-like membranes to external sides of membranes of cells deprived of PON1 activity in the mechanism mediated by the HDL scavenger receptor class B member 1 (SR-B1). PON1, which undergoes such a transition, maintains its enzymatic activity, and can thereby modify sensitivity to oxidative stress in the destination tissue [23]. PON1 has been determined to be widely expressed in tissues, such as the liver, kidney, heart, brain or intestine [24]. The lack of PON1 mRNA expression in these tissues supports the hypothesis that HDL molecules are likely to serve as carriers transferring PON1 to various tissues requiring its activity [25]. High PON1 expression is observed in epithelial cells, especially lung epithelium, due to its high exposure to a great range of xenobiotics [26].

PON1, due to its different activities, is able to hydrolyze an exceptionally wide variety of substrates: toxic oxons (metabolites of organophosphate compounds like parathion and chlorpyriphos used as insecticides); nerve gases (sarin, soman); aromatic esters such as phenylacetate and its several derivatives; lactones (both aromatic and aliphatic); and cyclic carbonates including dihydrocoumarin, γ-butyrolactone and homocysteine thioclatone (HCTL) [17]. The latter compound mentioned, a product of homocysteine enzymatic conversion, is known to interact with proteins through N-homocysteinylation. The following alteration in protein structures leads to changes in gene expression, redox transition impairment, amyloid formation and cytotoxicity. HCTL was suggested to induce inflammatory responses and oxidative stress in endothelial cells, leading to vascular dysfunction, and is related to cancer development and neurodegenerative diseases [27]. Given this, the HCTL molecule was proposed to be the physiological substrate for PON1 since it would be able to protect proteins from homocysteinylation due to its activity toward HCTL [28,29]. However, more recent research indicates that the HCTL-ase activity of PON1 has minimal physiological relevance due to low affinity and efficiency for this process, and the detoxification of HCTL is performed to a dramatically larger extent by the biphenyl hydrolase-like protein (BPHL) [25,30].

Notably, the identified substrates for PON1 are mostly unnaturally occurring compounds. Studies concerning the PON1 structure–activity relationship and experimental research focused on the exploration of possible in vivo substrates led to the conclusion that the native PON1 activity is most probably lactonase, as first stated by Khersonsky and Tafwik [15]. This finding remains in agreement with the knowledge that lactones are compounds widely distributed in plants. It corresponds with the assumption of the existence of some natural substances, which PONs have developed to regulate. The fact that all PONs are able to inactivate the quorum sensing factor of *Pseudomonas* bacteria, which is a derivative of the N-acyl homoserine lactone, and in this manner can prevent mammals from infection and lethality caused by *Ps. aeruginosa*, is supporting evidence as well. Furthermore, the latest research has shown that PON1 is active toward some naturally occurring δ-lactone eicosanoids: cycloepoxycyclopentenone (cycloEC) and 5,6 dihydroxy-eicosatrienoic acid lactone (5,6-DHTL), derived from arachidonic acid [31,32]. The hydrolysis of DHTL in the endothelial cells, to which PON1 was recently shown to penetrate, affects vascular dilation, causing vasoconstriction. Currently, PON1 lactonase activity is acknowledged to be preferential toward γ- and δ-lactones with long alkyl side chains. Additionally, estrogen esters are efficiently hydrolyzed by PON family members [33]. During studies concerning the possible antioxidant activity of the enzyme, it was found that PON1 associated with HDL, as well as purified PON1, is able to efficiently hydrolyze hydrogen peroxide—the major reactive form of oxygen produced in atherogenesis. Therefore, separate peroxidase activity, expressed in the neutralization of fatty acid hydroperoxides and cholesteryl ester hydroperoxides, is also distinguished [34].

### 2.2. Physiological activity

#### 2.2.1. Substrate Hydrolysis

Due to its hydrolytic activity toward a broad spectrum of substrates with different structures, PON1 is involved in the metabolism of various pharmaceuticals. In order to achieve the optimal activity and pharmacokinetic properties of a drug, a prodrug concept was developed—a compound of little or no pharmacological activity, which is, after administration, converted into the active drug via enzymatic and chemical reactions. The bioactivation of prodrugs is primarily performed by esterases, both intestinal and plasma, including PON1 whose activity toward pharmaceuticals is conditioned by presence of lactone or cyclic carbonate moieties in the drug molecule [35]. In 1998, PON1 was reported to rapidly and efficiently transform a novel broad-spectrum fluoroquinolone antibiotic prulifloxacin into its active form, ulifloxacin [36]. Apart from that, PON1 was, through N-terminal peptide sequencing, identified to be responsible for the in vivo bioactivation of angiotensin receptor blocker olmesartan medoxomil [37]. Notably, the LACase activity of PON1 turned out to be of value in topical glucocorticoid drug metabolism—as soon as drug molecules reach the circulation, they are inactivated by the enzyme. This allows the reduction of systemic effects of glucocorticoids, which are often undesirable, and limit the drug’s effects only to the site of application [38].

#### 2.2.2. PON1 as an Antioxidant

It is now well documented that oxidative stress is a result of the imbalance between free radical formation and their scavenging system in favor of the first. The impairment in antioxidant defense mechanisms is involved in the pathogenesis of numerous metabolic diseases, including atherosclerosis. In an accepted mechanism of atherosclerotic plaque development, the modification of LDL particles in the vascular endothelium plays a crucial role. Oxidative stress conditions enhance LDL binding to its receptors on macrophages, which leads to cellular oxygenase activation and LDL oxidation. An uptake of ox-LDL by macrophages via their CD36 scavenger receptor initiates the accumulation of cholesterol originating from plasma LDL. Such cholesterol-loaded macrophages transformed into foam cells characterize the atherosclerotic lesion in the arterial wall. It is accompanied by chronic low-intensity inflammation, and such conditions additionally accelerate the progression of the disease [39,40,41].

The hypothesis of PON1 being the HDL component responsible for its antioxidant, and therefore antiatherogenic, action was validated in the experiment on PON1 knockout mice. Not only were the animals devoid of PON1 more sensitive to insecticide-induced toxicity, but their HDL particles were also demonstrated to be unable to prevent LDL from oxidation in comparison to wild-type mice, becoming susceptible to oxidation themselves. PON1 knockout mice fed with a high-fat diet were also more susceptible to develop atherosclerosis [42]. Experiments on double PON1/apoE knockout mice confirmed that PON1 protects lipoproteins from oxidative damage and preserves their proper functions [34]. Basically, studies on animal models have shown that PON1 is a major agent reducing the atherogenicity of lipoproteins owing to its lipolactone-hydrolysing activity [43]. This gave rise to numerous studies aiming to elucidate the mechanisms of PON1 antiatherogenic activity, owing to which it is now known that PON1 contributes to the atheroprotective activity of HDL in several manners.

**Figure 1:**
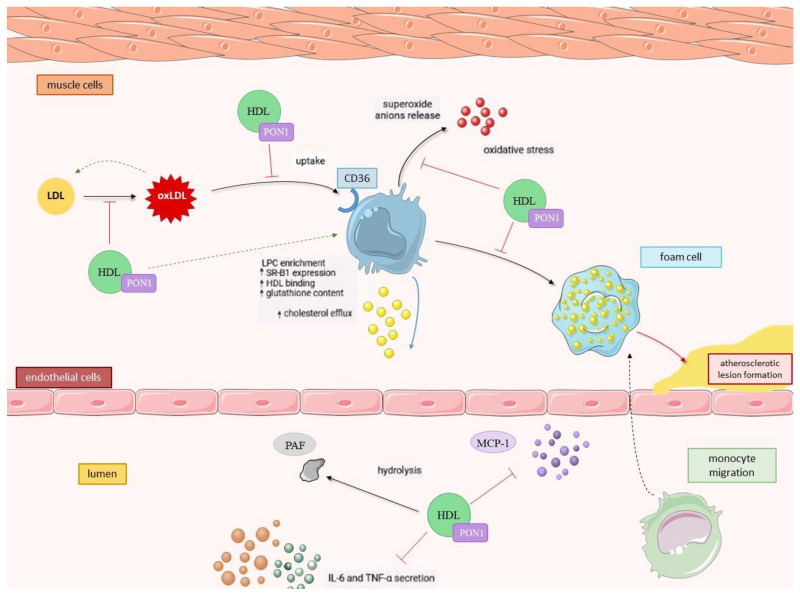
Schematic presentation of multifaceted PON1 antioxidative, antiatherogenic and anti-inflammatory activity. PON1 antioxidant activity is related to the suppression of oxidized LDL (ox-LDL) formation and uptake by macrophages via their CD36 scavenger receptors, which otherwise would lead to foam cell development. Other than that, PON1 contributes to atheroprotective activity of HDL in several other manners: stimulates macrophage cholesterol efflux, enriches macrophage plasma membrane with lysophosphatidylcholine (LPC), increases scavenger receptor class B type 1 (SR-B1) expression and HDL binding. The anti-inflammatory activity is related to platelet activating factor (PAF) hydrolysis by PON1 and inhibition of pro-inflammatory cytokines secretion, such as tumor necrosis factor-α (TNF-α) and interleukin-6 (IL-6). PON1 is also able to inhibit monocyte migration to subendothelial space due to its interaction with macrophage chemoattractant protein-1 (MCP-1). Figure created with the use of Servier Medical Art by Servier.

To start with, PON1 has been proven to stimulate HDL-mediated macrophage cholesterol efflux via the ATP-binding cassette transporter ABCA1 in a direct interaction. Although ApoA1 is able to induce such an effect as well, its minor extent and marginal importance implies that PON1 activity is crucial and determines the efficacy of this process [44]. Secondly, the hydrolytic activity of PON1 associated with HDL particles toward the macrophage plasma membrane surface of phospholipids results in lysophosphatidylcholine (LPC) formation, and the rate of generated LPC was observed to positively correlate with the activity of the PON1-HDL complex. Importantly, LPC was determined to inhibit cholesterol biosynthesis in liver cells at the stage of lanosterol to cholesterol transformation. It can be thereby concluded that PON1 has a direct inhibitory influence on cholesterol biosynthesis [45,46]. There is an additional effect of enriching macrophages with LPC as it enhances HDL-macrophages binding, contributing to the overall improvement of the HDL-mediated cholesterol efflux rate via the enzymatic conversion of macrophage membrane phospholipids to LPC [44] (Figure 1.).

Furthermore, it was previously discovered that oxidized LDL (ox-LDL) under oxidative stress conditions is able to induce the expression of the CD36 scavenger receptor and thereby enhance its own cellular uptake by macrophages, which is a step toward atherosclerotic foam cell formation. Here, PON1 does play a role—the specific hydrolysis of lipid peroxides performed by this enzyme reduces the rate of macrophage ox-LDL uptake, inhibits macrophage superoxide anion release and increases cellular glutathione content [47,48]. PON1’s ability to increase HDL-macrophage binding and its protective activity toward macrophage-mediated LDL oxidation could not be possible unless PON1 could bind to cells. Indeed, PON1 has been proven to bind to their plasma membrane, presumably through cellular phospholipids, and the binding sites were discovered to be common to PON1 and HDL. After being bound, PON1 undergoes internalization and accumulates in the macrophage’s cytosol, where it remains biologically active [49].

#### 2.2.3. Anti-inflammatory Activity of PON1

Along with being an antioxidant, PON1 is acknowledged as an anti-inflammatory factor. Its activity is targeted toward macrophage chemoattractant protein-1 (MCP-1)—a major chemokine involved in the recruitment of monocytes to the subendothelial space—and the migration to the inflammation site where they differentiate into macrophages and initiate foam cell formation. For this to occur, the interaction between the chemokine (C-C motif), ligand 2 (CCL2) and chemokine receptor (CCR2) is crucial. PON1 was observed to attenuate the endothelial secretion of MCP-1 by downregulating its expression [50,51]. Another pro-inflammatory factor, the anti-CD54 monoclonal antibody known as intercellular adhesion molecule-1 (ICAM-1), was found to be suppressed by PON1 as well. The incubation of endothelial cell lines with LDL oxidized in the presence of PON1 reduced the expression of ICAM-1 on the cell surfaces by 75% in comparison to LDL oxidation proceeded by no addition of PON1 [52]. Additionally, Mackness et al. [53] have shown that PON1 is able to hydrolyze the platelet activating factor (PAF)—a pro-inflammatory mediator—which stimulates monocytes to migrate and transform into macrophages, likewise to MCP-1. Moreover, PON1 was observed to inhibit pro-inflammatory cytokine secretion, such as tumor necrosis factor-α (TNF-α) and interleukin-6 (IL-6), in stimulated macrophages via increased SR-B1 expression, whose mechanism is also involved in HDL-mediated macrophage apoptosis prevention and serves as an alternative pathway in cholesterol efflux [54]. Additionally, the expression of CD36 and integrin CD11b was found to be suppressed due to PON1 activity [55].

### 2.3. PON1 Status in Disease Conditions

**Figure 2:**
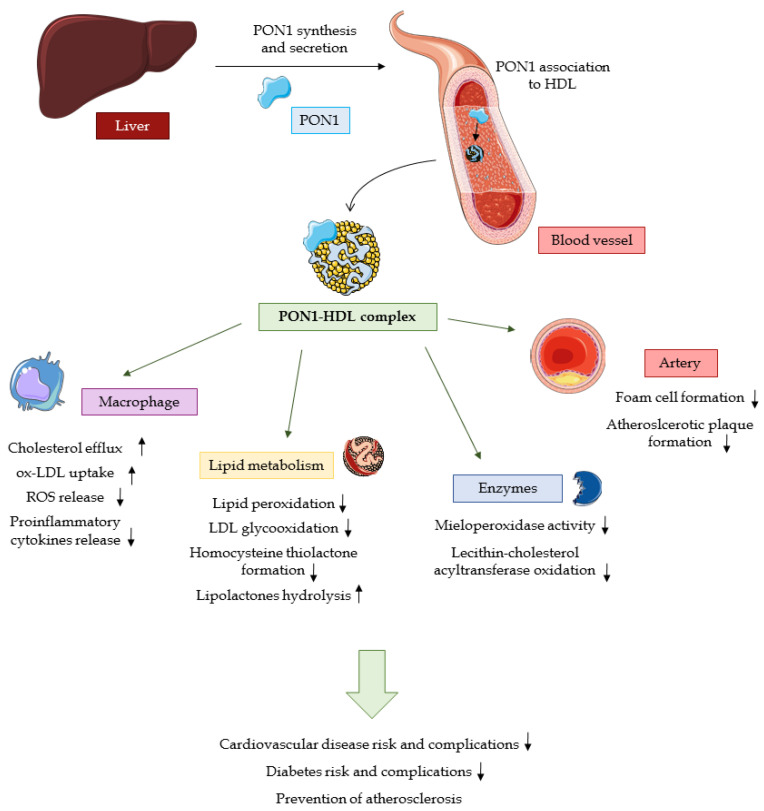
A schematic representation of PON1-HDL complex, formed after PON1 had been synthesized in the liver and secreted to the bloodstream, functions in overall cardiovascular risk reduction and disease prevention. Figure created with the use of Servier Medical Art by Servier.

The disruption of the oxidant–antioxidant balance in favor of reactive oxygen species (ROS) formation is typical for numerous non-communicable diseases and is also believed to contribute to their etiology. The excessive amount of ROS and lipid peroxides generated in oxidative stress conditions bind to the active site of PON1 molecules, which is no longer available for its other substrates. Such a saturation of the enzyme pool by increased amounts of lipoperoxides results in its decreased activity [56], and this loss in PON1 activity is indeed observed in the course of oxidative stress-related diseases, such as cardiovascular diseases (CVD), diabetes, liver diseases, cancer, immune diseases or some neurological disorders. Decreased PON1 activity has been correlated to a high risk of CVD and predestines type 2 diabetic patients to heart failure [57,58] since this enzyme contributes to the prevention or decrease in the rate of cardiovascular complications associated with type 2 diabetes via numerous mechanisms, i.e., the reduction of plasma oxLDL and lipid hydroperoxide levels, inhibition of myeloperoxidase activity, reduction of foam cell formation, suppression of macrophage proinflammatory responses and their ability to release ROS with an enhancement of macrophage cholesterol efflux, some of which have been described in detail in previous paragraphs of this section. PON1 also plays a role in the prevention of the oxidative inactivation of lecithin-cholesterol acyltransferase enzyme (LCAT) and counteracts glucose-induced glycooxidation of LDL [58]. The contribution of the PON1–HDL complex to CVD, diabetes and atherosclerosis prevention is summarized in Figure 2. Lowered serum PON1 levels have been acknowledged independent risk factors for acute coronary events [59]. As the prevalence of CVD increases steadily and is still a main cause of mortality worldwide [60], its interrelation with PON1 activity has been comprehensively reviewed, and the association of CVD with genetic polymorphisms of PON1 has been studied and reviewed as well [61,62]. Kotur-Stevuljević [63] reviewed the state of knowledge concerning relationships between PON1 and atherosclerosis-related diseases.

The involvement of PON1 in neurodegenerative diseases has also been reported; its decreased activity is associated with ischemic stroke, amyotrophic lateral sclerosis, various types of dementia, Alzheimer’s disease and Parkinson’s disease [64,65]. Salari et al. [66] in their systematic review identified PON1 as a potential factor in multiple sclerosis pathogenesis. In the case of Parkinson’s disease, it is the accumulating dopaminergic neurotoxin 1-methyl-4-phenyl-tetrahydropyridine of the chemical structure, similar to OPs, which interacts with PON1 [65]. Some new reports are available concerning the role of PON1 in non-alcoholic fatty liver disease [67], psoriasis [68] or chronic kidney disease [69]. A recent meta-analysis [70] has shown that the POase and AREase activities of PON1 are decreased among patients suffering from chronic obstructive pulmonary disease (COPD) when compared to healthy control group. It has been observed that the severity of COPD plays a role—interestingly, mild and moderate COPD is characterized by slightly lower PON1 activities than the severe grade of this disease. This can be explained by—decreasing progressively in the course of the disease’s parenchyma destruction—contact with myeloperoxidase, a strong PON1 inhibitor released by immune cells infiltrating alveoli.

Tumorigenesis is also closely linked to excessive ROS formation, as it is believed to be oncogenic—elevated ROS levels promote genetic instability via oxidative DNA damage and serve as signaling molecules, activating molecular pathways connected to increased cancer cell survival and silencing tumor suppressor genes [59]. A number of recent studies investigating the relationship between PON1 and cancers showed low expression and activity of this enzyme. The decrease in all PON1 activities has been shown to range between about 20 and 80% in various types of cancer (e.g., lung, breast, prostate, gastrointestinal, central nervous system tumors and several types of lymphomas), where the loss of antioxidant protection is manifested in increased amounts of hydroperoxides, ox-LDL, malondialdehyde and conjugated dienes [71]. Increased susceptibility to some cancers is suspected to be related to PON1 polymorphic variants [72]. It was suggested that cancer cells are able to utilize PON1’s capability to penetrate cells in its active form as one of the apoptosis resistance mechanisms in neoplastic transformation processes [73]. The decreased activities of PON1 in cancer might also be connected with the modification in protein glycosylation, affecting PON1, which is a glycoprotein. The signaling pathways of mitogen-activated protein kinases (MAPK)/extracellular signal-regulated kinases (ERK), protein kinase C (PKC) and p44/42, which control cell growth and differentiation, apoptosis and angiogenesis, are also involved in PON1 regulation [72].

The latest study in renal cell carcinoma has revealed that its progression was associated with the hypermethylation of the PON1 gene, while its demethylation significantly decreased the proliferation, migration and invasion of cancer cells [74]. Additionally, an increase in the histone deacetylase activity that accompanied the decrease of PON1 activity observed in colorectal cancer progression suggested that histone deacetylase contributes to the loss of PON1 activity during cancer development, but the exact mechanism is yet unclear [75]. Huang et al. [76] reported an inverse correlation between the PON1 expression in hepatocarcinoma cells and the status of vascular invasion. The authors put forward the idea of PON1 as a biomarker of microvascular invasion, the diagnostic value of which was assayed and recognized as potentially useful in introducing personalized treatment strategies [77].

Recently, the correlation between PON1 activity with the risk of cancer recurrence after radiotherapy (RT) has been investigated for prostate cancer (PCa) patients [78]. The prospective study has been conducted on 56 men suffering from PCa subjected to RT, among which 11 experienced a recurrence confirmed by biopsy. The statistical analysis of data obtained during a follow-up period lasting up to 84 months has shown a significantly higher activity of PON1 in patients with the cancer recurrence after RT in comparison to recurrence-free subjects, which suggests that the measurement of PON1 activity can provide valuable information as a tool in predicting cancer recurrence after RT; the accuracy of which could exceed currently used predictive models constructed with the use of patients’ clinical records. Certainly, further studies, including more patients along with longer follow-ups, are necessary in order to verify this hypothesis.

Apart from cancers, coronavirus disease 2019 (COVID-19) remains one of the greatest present-day health concerns since December 2019. Naturally, severe acute respiratory syndrome coronavirus-2 (SARS-CoV-2) which causes COVID-19 has become a subject of extensive research worldwide, and some studies focused also on PON1 activity in the context of this disease. Begue et al. [79] found that PON1 is less abundant in HDL molecules isolated from patients who suffered from COVID-19, and HDLs themselves possess some quantitative and qualitative abnormalities, such as enrichment in acute phase proteins and decreased Apo-A1 levels. A study by Cho [80] provided similar results, as HDLs isolated from severe COVID-19 patients were deprived of their protective effect on endothelial cells and displayed a great loss in anti-inflammatory and antioxidant activity. Preliminary data obtained from a small, hospitalized group by Rodríguez-Tomàs et al. [81], confirmed later by a larger study involving over 1300 subjects divided into study and control groups [82], have shown that serum PON1 AREase activity in COVID-19 patients drops to about half the value characteristic for healthy individuals. The concurrent increase in PON1 concentration is in all likelihood related to the upregulation of its synthesis in order to balance its insufficient enzymatic activity. Additionally, the reduction in PON1 activity occurred independently of the severity of the disease. Therefore, the authors have stated that the measurement of PON1 AREase activity in serum samples could be a useful diagnostic, but not prognostic, marker of COVID-19 occurrence—it has been proposed to be a simple and inexpensive tool in COVID-19 community-based diagnostics as an alternative to gold-standard detection of the viral RNA by nucleic acid amplification tests, especially in low-income countries with low levels of vaccination. Although, there are some obvious limitations to the applicability of this concept, of which lack of specificity would be the key one. It has been mentioned that similar, lowered levels of PON1 activity are found in numerous diseases. Other contributing factors, such as medications taken or exposure to tobacco smoke, can also modify PON1 activity and thereby influence the measurement result. Nevertheless, since high PON1 activities are found only in individuals not infected with SARS-CoV-2, such a determination of its activity could be considered an initial communal screening method.

## 3. Modifiable Factors Impacting Human PON1 Activity

### 3.1. Environmental Factors Influencing PON1 Activity

#### 3.1.1. Organophosphates (OPs)

Organophosphates, which are insecticides known since the early 20th century, are basically triesters of phosphoric acid but, as main components of nerve gases, have also been used in warfare as a weapon against military targets and civilians. Currently, OP pesticides are extensively applied in agricultural and industrial purposes, and their use results in a high rate of exposure, especially among inhabitants of rural areas and agricultural industry workers. OPs can be absorbed through the skin or by inhalation or ingestion of contaminated food and water [83].

OP compounds are lipophilic; therefore, they undergo quick absorption and distribution to the tissues. Toxicodynamic and toxicokinetic characteristics of OPs can be attributed to their biochemical features, enabling interactions with hydrolases. Most OP insecticides are organothiophosphates. As only OPs possessing P=O moiety are able to interact with AChE or neuropathy target esterase, thus, a metabolic transformation to corresponding oxygen analogs via numerous P450 isozymes is required to turn them into active forms [84]. The direct toxic effect is exerted through the inhibition of acetylcholinesterase (AChE) in the central nervous system. This results in the accumulation of acetylcholine (ACh), which is not efficiently hydrolyzed, at cholinergic synapses and the hyperstimulation of muscarinic and nicotinic cholinergic receptors. The rate of AChE inhibition reaching 60–70% in acute OP poisoning is manifested by muscle fasciculations, weakness, parasympathetic symptoms (emesis, bradycardia, hypotension) and anxiety, eventually leading to eventual depression of the respiratory control center and death on the minutes to several hours scale, known as the cholinergic crisis [83]. The intermediate syndrome, occurring hours to days after the cholinergic overstimulation, includes myocyte necrosis, the downregulation of postsynaptic AChE receptors and inhibition of postsynaptic ACh release [85]. Neurotoxic effects are among delayed and late symptoms related to repeated exposures of low levels of OPs; however, it was deduced from the multiple lines of evidence that some other, non-cholinergic mechanisms must be involved in the development of these health effects, which cannot be attributable to AChE inhibition. A considerable, still growing number of recent reports implicates the contribution of oxidative stress in both acute high-level poisoning and long-term exposures [86]. In many studies, elevated levels of oxidative stress markers were found in individuals exposed to pesticides and nerve gases. Due to high levels of polyunsaturated fatty acids susceptible to oxidation present in the brain, along with high oxygen utilization in brain tissue, this organ is particularly sensitive to oxidative damage. The intensified generation of ROS induced by OPs triggers cholinergic receptor activation and a following glutaminergic transmission stimulation via N-methyl-D-aspartate (NMDA) receptors, which further accelerates oxidative reactions via Ca^2+^ dependent enzymatic cascades. Moreover, oxidative attacks disrupt the functionality of neuronal mitochondria, raising the rate of apoptosis, which is directly connected to the pathogenesis of numerous neurological disorders [87]. It emphasizes the role of PON1 hydrolytic activity in detoxifying OP compounds as it is a major protective factor against their neurotoxicity once OPs enter the circulation [88].

PON1 is referred to as a biomarker of susceptibility to OP toxicity in vertebrates and is considered to be its major determinant. The catalytic efficiency of PON1 toward specific oxon substrates assayed in vitro can serve as a predictor of its in vivo protection level [84]. Polymorphic variants of PON1 investigated in numerous epidemiological studies throughout the past 30 years seem to be relevant in the modulation of individuals’ sensitivity to OPs as they differ in detoxification efficiency of specific OP compounds [89]. Many of the studies lack measurements of PON1 concentration and activity, as attention was devoted mainly to SNP identification and elucidation, and these cannot be acknowledged as comprehensive [90]. On the contrary, an inverse relationship—i.e., a direct influence of OPs on PON1—was not extensively studied. Some new light on this issue was shed by studies on human hepatocellular carcinoma (HepG2) cell lines treated with methyl parathion and chlorpyriphos, where a significant decrease in PON1 mRNA expression was noted, along with the increased secretion of pro-inflammatory mediators [91,92]. The molecular basis of these findings has been elucidated as the downregulation of PON1 gene expression either via the cytokine-mediated modulation of the PON1 promoter or through the farnesoid X receptor (FXR)-mediated pathway, suppressing PON1 transcription hepatic nuclear factor. The other proposed mechanism of PON1 gene modulation driven by OPs is the downregulation of peroxisome proliferator-activated receptor family (PPAR) genes, and in consequence, the decrease of PON1 synthesis [92]. PPARs comprise a subfamily of nuclear receptors, binding in the form of heterodimers to response elements in target genes, often involved in the control of metabolic processes as well as oxidative stress and inflammatory responses. Moreover, it has been noticed that the activation of the PPAR-dependent signaling pathway results in the downregulation of MCP-1 expression. It indicates that PPARs are an essential component in the regulation and coordination of both PON1 and MCP-1 expression [93].

#### 3.1.2. Heavy Metals

Contemporarily, the high level of pollution with metal contaminants is among the major environmental concerns and is also a threat for human beings. Epidemiological studies indicate that the widespread presence of heavy metals causes chronic, low-dose exposure that is very common and no longer limited to occupational risk. Xenobiotic metals (i.e., having no biological role in the human body at any dose, or biologically relevant but in a toxic dose, like copper) were linked with numerous health problems, particularly cardiovascular diseases, atherosclerosis and dyslipidemia [94,95]. Some of them are referred to as heavy metals, including mercury, nickel, cadmium and lead, but also some rare earth metals, such as cerium or lanthanum, are known to inhibit PON1 activity since the 1950s with half maximal inhibitory concentration (IC_50_) values in sub-micromolar range [4,96]. In 2010, metallic ions such as Cr^2+^, Fe^2+^ and Zn^2+^ were included in the list of PON1 inhibitors [95].

Overall, the toxicity of heavy metals such as cadmium, mercury, arsenic and lead is attributable to their catalytic activity toward the oxidation of biomacromolecules and the following DNA and tissue damage in oxidative stress conditions. Further, prolonged heavy metal exposure leads to the depletion of glutathione (GSH) resources and the inactivation of certain antioxidant enzymes containing sulfhydryl groups, including PON1. It applies to three PON1 cysteine residues, the modification of which is likely to impair PON1 activity [97]. In the example of zinc and nickel, Josse et al. [98] indicated that several metals may bind to histidines (His-115 and His-134) in positions essential for PON1 function and therefore decrease its activity through direct interaction with the protein. Studies on lead battery manufacture workers [99] suggest that, in the case of lead exposure, calcium-binding sites of PON1 are affected. The concentration of lead in the blood of research participants was in the low micromolar range at the levels assessed to inhibit PON1 activity ex vivo. On the other hand, Laird et al. [100] connected the loss of PON1 activity to modifications in copper utilization by lead intoxication, as Cu^2+^ deficiency is involved in a decrease of PON1 activity. In the case of methylmercury, most studies on different populations demonstrated that exposure is negatively correlated with PON1 activity—Hg ions are able to inhibit the DNA-binding activity of transcription factor Sp1 (specificity protein 1) located in the PON1 gene promoter via interaction with Cys2His2 zinc-binding domains, which downregulates PON1 expression [101].

### 3.2. Influence of Diet and Nutritional Habits on PON1 Activity

#### 3.2.1. Dietary Lipids

Dietary factors, to a similar extent as those previously described, are able to modulate PON1 by direct interaction (inhibition/activation) or in terms of its synthesis, secretion, stability and association to HDL modifications, as well. Particularly, the amount and type of lipids consumed are essential regulatory factors of PON1. For instance, a high-fat diet with high amounts of cholesterol is related to an increased secretion of inflammatory cytokines in the intestine, which is followed by ROS formation and inflammatory response. Leukocyte infiltration into the hepatic tissue intensifies oxidative stress and lipid peroxide production, which is able to downregulate PPAR gene expression, and therefore PON1 expression is also downregulated. It may be followed by either the inhibition of PON1 gene expression, which directly leads to a decrease in PON1 secretion, and/or the reduction in ATP-binding cassette subfamily A member 1 (ABCA1) gene expression, which consequently reduces the level of HDL synthesis. The ultimate outcome of both pathways is a decrease in PON1 activity [102]. Likewise, a high intake of oxidized lipids present in thermally stressed fatty acids contributes to the increased secretion of oxidized chylomicrons, which enhances the synthesis of ox-HDL in the liver. Such a modification lowers HDL’s ability to associate with PON1, which is reflected in the decreased activity of PON1 in comparison to the activity of PON1 complexed by unaffected HDL particles. It was demonstrated in a study on healthy men, whose dietary saturated fat was replaced with trans-fat [103].

Monounsaturated fatty acid (MUFAs) intake has been reported to increase PON1 activity in several independent studies. Cherki et al. [104], with the use of virgin argan oil and extra virgin olive oil, provided evidence that enriching LDL with antioxidants present in these oils can decrease their susceptibility to lipid peroxidation and stabilize PON1 structure, preserving it from oxidation. It is in line with the results of Nguyen et al. [105], who proved that oleic acid and oleoylated phospholipids exert a protective influence on PON1. According to the authors, it could be possible either due to the resistance of membrane phospholipids enriched in oleyl groups to oxidative modification or the enhancement of very high-density lipoprotein particle formation. Oleic acid intake was shown by Tomás et al. [106] to positively influence PON1 activity, and the effect was particularly beneficial in the subjects who possessed the R allele of the PON1 192 polymorphism. Likewise, in HDL isolated from serum enriched with di-oleoyl-phosphatidylcholine, all three activities of PON1 were found to be significantly increased [107]. Polyunsaturated fatty acids (PUFAs), on the other hand—despite their favorable effect on inflammation via the inhibition of Toll-like receptors TLR2 and TLR4 and the subsequent inactivation of the NF-κB pathway, whose activity leads to inflammatory cytokine secretion, and insulin-sensitizing effects due to an increase in leptin secretion—were shown to decrease PON1 activity [108,109]. This might arise from PUFA’s susceptibility to lipid peroxidation. However, there is still some inconsistency in the results of studies concerning PUFAs—according to Calabresi et al. [110], the consumption of ω-3 PUFAs by a group of individuals with familial combined hyperlipidemia resulted in an increase of postprandial serum PON1 levels.

#### 3.2.2. Glucose Intake

PON1 activity has been proven to be lowered in patients suffering from both type 1 and 2 diabetes (T2D) compared to healthy controls in numerous studies [111,112], and this effect was identified as not dependent on the PON1 genotype. It is well established that the state of hyperglycemia in cells induces the non-enzymatic glycation of proteins, resulting in the formation of Schiff bases and advanced glycation end products (AGEs). Therefore, the direct PON1 active site lysine modifications via glycation has been proposed as one of the mechanisms leading to the loss of its catalytic activity in hyperglycemic conditions [113]. Hedrick et al. [114] calculated that the loss in purified PON1 after incubation with 25 mM glucose reached 40% and observed that glycated PON1 did not prevent monocyte adhesion to endothelial cells.

HDL-bound apoproteins may be subjected to glycation modifications as well. The excess of glucose triggers intensified lipid peroxidation and HDL oxidative modification, leading to compositional changes and alterations in the apoprotein structure. It translates to PON1’s decreased activity as a complex with HDL—these conformational changes negatively affect PON1 interactions with phospholipids and apoproteins at the lipoprotein surface. Additionally, it has already been pointed out that lipid peroxidation products inactivate PON1 in a direct mechanism, and lower protection levels entail more extensive oxidative damage [115].

In vitro studies conducted by Rosenblat et al. [116] indicate that glucose inactivates both free and HDL-bound PON1 in a dose-dependent manner and is able to induce an additional dissociation of PON1 from HDL particles, destabilizing the complex. POase and AREase activities of PON1 seem to be inhibited in the direct inactivation mechanism, while the significant reduction in LACase activity is connected to glucose-induced loss of HDL’s capability to enhance cholesterol efflux from macrophages (outlined in Paragraph 2.2.2.). In addition, in diabetic patients—unlike healthy subjects—most PON1 activity was exhibited in the lipoprotein deficient serum (LPDS) fraction instead of HDL particles [117]. Based on ex vivo studies with diabetic individuals in comparison to healthy controls, it can be concluded that high glucose concentrations inactivate both HDL-bound and free PON1 [116].

On a molecular basis, it is suspected that high diacylglycerol levels occurring in hyperglycemic states cause PON1 gene transactivation via the PKC-mediated pathway, although conflicting results concerning this issue have been obtained. Ikeda et al. [118] studied the regulation of PON1 synthesis in hepatic cell cultures. However, their findings were not consistent with the literature reports as they observed upregulation of PON1 gene transcription by the glucose mediated by Sp1 and PKC-related pathway, which occurred in a dose-dependent manner. It should be kept in mind that transcriptional modulation in diabetes in vivo is supposedly much more complex, and exact conditions, such as chronic microinflammation, hyper- or hypo-insulinemia and ROS accumulation, which might affect PON1 expression, have not been successfully reconstructed in vitro. Additionally, the study was short-term, and further, prolonged research is necessary.

#### 3.2.3. Plant-derived Compounds

The most widely studied chemicals in PON1 activity assessment were undoubtedly polyphenols—a vast heterogenous group of biologically active compounds with at least one aromatic ring in their chemical structure, widespread among plants and naturally present in fruit, vegetables and other food products of plant origin. Regulatory effects demonstrated for polyphenolic compounds, both flavonoid and non-flavonoid, include prevention from metabolic syndrome, endothelial dysfunctions and cardiovascular diseases due to their antioxidant and anti-inflammatory activity [119], which drew attention to the possible involvement of PON1 in their mechanisms of action. Hence, a number of human studies on how plant-derived compounds may participate in PON1 activity modulation have been carried out.

Pomegranate juice, a source of numerous bioactive components of strong antioxidant character, was particularly often used in studies. Along with PON1 concentration and activity, lipid parameters and malondialdehyde (MDA) as an oxidative stress marker have been assessed. Rock et al. [120] demonstrated an increase in enzymatic activity of PON1, whose association with HDL has been enhanced and stabilized, and oxidative stress markers were shown to be reduced after a 6-week period of pomegranate juice intake. A significant increase in PON1 AREase and POase activity and positive correlation between PON1 activities and total HDL concentration, but with no changes in both HDL and PON1 concentration in serum, was observed, remaining in agreement with the previous findings and confirmed in further studies [121,122,123,124]. The authors claim that the rise in PON1 activity is related to tannin and anthocyanin content of the consumed juice. Wu et al. [125] demonstrated on a group of hemodialysis patients that a 6-month supplementation with 1 g of purified pomegranate extract raises PON1 LACase activity but causes no changes in POase and AREase activities.

In addition, Fuhrman et al. [126], using recombinant human PON1, discovered that polyphenols of pomegranate juice enhance the binding of PON1 to HDL in vitro and in diabetic patients. Moreover, the polyphenolic compounds, such as quercetin, elagitannins and—mostly—resveratrol, were proved to mediate the upregulation of PON1 mRNA transcription in hepatocytes from the cell line HuH7 via the PPAR-γ activation and PKA-cAMP signaling cascade, which leads to the increased secretion of biologically active PON1. Reliable clinical and experimental evidence indicates that the activation of PPARs, particularly PPARγ, increases PON1 expression via PON1 gene upregulation. The other proposed mechanism of PON1 upregulation is via the activation of the ligand-activated transcription factor through the aryl hydrocarbon receptor (AhR) and its binding to xenobiotic responsive elements (XREs) within the PON1 promoter. The modulation of the PON1 transcription can also be based on the interaction of sterol regulatory element-binding protein-2 (SREBP-2) with the sterol responsive element-like sequence on the PON1 promoter, linked to MAPK signaling cascade. This ultimately results in PKC activation. Polyphenolic compounds can mediate both these pathways [127,128,129,130].

Resveratrol was shown in several independent studies on hepatocyte cultures and hepatoma cell lines to significantly increase PON1 activity and has been reported to induce PON1 mRNA expression and activity in a human cohort (reviewed in [129]). Additionally, quercetin has been proven to upregulate PON1 gene expression, as confirmed by Garige and coworkers [131]. This effect has been attributed to the PON1 translocation to the cell nucleus and its subsequent interaction with the sterol responsive element-like sequence. The upregulation of PON1 can also be induced by berberine via c-Jun N-terminal kinase (JNK)-mediated c-Jun phosphorylation and its binding to AP-1 sequence, which promotes PON1 expression, as observed by Cheng et al. [132] on two hepatic cell lines. Recombinant PON1 has been reported to physically interact with an isoflavane glabridin, and it resulted in an improvement in the enzyme’s antioxidant efficacy toward ox-LDL cholesteryl ester hydroperoxides [133]. Although several in vitro studies indicated that genistein, an isoflavone compound, is a potent inducer of PON1 activity, human studies failed to prove this correlation [134]. In a study on post-menopausal Korean women, after supplementation with genistein combined with polysaccharides, PON1 activity remained unchanged, but the activity of a different antioxidant enzyme, glutathione peroxidase, had been significantly increased [135]. Therefore, it was concluded that such an intervention improves antioxidant status, but it occurs in a PON1-independent manner. Regarding human studies, a significant increase in PON1 activity was observed in hemodialyzed patients receiving tea catechins, known for their antioxidant function and preventive properties from cardiovascular diseases [132], and in hypercholesterolemic subjects as a result of anthocyanin intake [136]. Apart from tea, there are other sources of catechins since representatives of this group are present; for instance, they are also in peaches, grapes, wine and vinegar, along with other bioactive compounds such as procyanidins and anthocyanins, and these have also been demonstrated to upregulate PON1 levels and increase its protective activity against lipid peroxidation [137]. In addition, the latter has been observed to increase HDL levels and cholesterol efflux capacity with a reduction of HDL oxidation, suggesting their usefulness as cardioprotective nutraceuticals [138].

Curcumin, a commonly used spice of Eastern cuisine and a polyphenolic compound sourced from the *Curcuma longa* root, was demonstrated to cause PON1 transactivation in the HuH7 cell line in a dose-dependent manner [139] and induce PON1 in diabetic rats [140], although studies failed to reproduce this effect in the liver of curcumin-fed mice [139]. After analyzing the experimental data published in 47 research articles, Ganjali et al. [141] concluded that the activation of antioxidant enzymes, including PON1, is an important mechanism of action for curcumin enabling its protective effect on HDL function, associated with hyperglycemia mitigation and a decrease in the risk of atherosclerosis development. Among other substances with acknowledged PON1-inducing effects, betanin, isothiocyanates or licorice polyphenols can be listed [19], but the exact mechanisms in which these compounds affect PON1 are still to be explained.

#### 3.2.4. Vitamins and Trace Elements

Several studies report a role of vitamins in PON1 activity modulation. Although vitamin E is an acknowledged antioxidant, the studies failed to connect this beneficial effect to an increase in PON1 activity. Navarro-García et al. [142] reported that the AREase activity of PON1 decreased as a result of tocopherol consumption. Similarly, another study with healthy volunteers supplemented with 4100 IU of α-tocopherol has shown a drop in the AREase activity of PON1 associated with HDL_2_ and HDL_3_ for about 15% [143]. On the other hand, Tsakiris et al. [144] reported an increase in AREase activity among basketball players supplemented with α-tocopherol after exercise. It was seen to be otherwise in the case of vitamin C (ascorbic acid), which has been demonstrated to increase PON1 activity [145]. Interestingly, vitamin C was able to counteract the decrease in PON1 activity resulting from heat stress, and this effect was potentiated when combined with folic acid [146].

Studies focused also on the influence of B-vitamins on PON1 activity, but the results are contradictory. Navarro-García et al. [142] found that vitamins B1, B2 and B6 decrease PON1 POase activity, while different groups reported an increase in POase and AREase activity caused by vitamin B6 in diabetic rats [147]. Manolescu et al. [148] investigated the influence of the nutritional supplement ALAnerv^®^ containing B-vitamins and several minerals, such as selenium or calcium, with the addition of w-6 polyunsaturated fatty acids in a small group of post-acute patients. The results of this pilot study after a two-week intake of this product showed an increase in PON1 LACase activity, and therefore suggest that supplementation could be beneficial in improving the function of PON1. However, since the tested product consists of multiple components, this effect cannot be attributed to the particular compound(s).

The antioxidant properties of trace elements such as zinc, selenium, manganese or copper are widely known [149]. Zinc supplementation has been shown to improve PON1 activity in hemodialysis patients in a randomized clinical study [150]. These results are supported by a study concerning PON1 related variables in patients with lower extremity artery disease, which reported a positive influence on serum PON1 activity and indicates that Zn can be protective against non-coronary atherosclerosis [151]. In a study evaluating serum concentrations of trace elements in obese women, the levels of several elements (e.g., Ca, Cu, Mg and Se) were significantly correlated with serum PON1 POase or LACase activity [152]. Begcevic et al. [153] demonstrated that the supplementation of cranberry extract with Zn and vitamin C enhance PON1 POase activity. Still, studies concerning the influence of minerals alone in healthy individuals are lacking.

### 3.3. Alcohol Consumption

Several studies have reported that moderate alcohol intake has a positive influence on HDL level and PON1 activity [154,155]. This can be explained by the effect exerted by ethanol on PKC, which, via Sp1 phosphorylation, modulates its binding to the PON1 upstream promoter region and thereby increases PON1 expression [90]. On the other hand, excessive amounts of alcohol consumed lead to PKC overexpression, which in turn downregulates PON1 and contributes to a loss of its catalytic activity. In chronic alcoholics, the decrease of serum PON1 activity reaches 50%, up to 70% in those who suffer from liver cirrhosis, as reported by Marsillach et al. [156]. The level of PON1 activity loss estimated by Rao et al. [155] was similar (45%). Moreover, a high intake of alcohol is known to activate P450 cytochrome enzymes and in a cascade of following reactions contributes to excessive ROS formation and the following lipid peroxidation [157].

Wine, particularly red and dry, is a unique alcoholic beverage rich in components of antioxidative character, among which flavonoids, including epicatechin, myricetin, quercetin and resveratrol, or anthocyanins with recognized antioxidant capacity, play a major role. It was previously demonstrated that red wine consumption enriches plasma LDL with flavanols (mainly quercetin), strong free radical scavengers. White wine, characterized by lower quantities of flavonoid compounds, would have a far weaker protective effect against LDL oxidation [39]. It is well accepted that a moderate amount of wine improves blood pressure and serum glucose values, modulates endothelial function and levels of HDL and LDL cholesterol, altogether lowering cardiometabolic risk factors [158]. Phenolic compounds have anti-inflammatory and antioxidant properties and play a role in tissue repair. The positive effect of red wine on PON1 modulation is in part dependent on ethanol, as mentioned at the beginning of this paragraph, but largely is due to the presence of resveratrol. The exact effect exerted by resveratrol on PPAR-γ receptors in terms of PON1 upregulation was described in Paragraph 4.1.3 of this manuscript. Despite these protective effects, it has to be kept in mind that the amount of consumed wine is of great importance since a chronic, excessive intake reverses the benefits into harmful response, leading to oxidative stress, endothelial dysfunction and increases risk of cardiovascular disease [155].

### 3.4. Cigarette Smoking

As for the influence of tobacco smoking on PON1 activity, the results of studies are consistent. It is widely known that smokers are more susceptible to developing cardiovascular diseases or insulin resistance. Smoking has been acknowledged as an independent risk factor for coronary heart disease [159]. Alterations in the lipid profile of smokers shall comprise an increase in free fatty acids levels in serum, increased synthesis of VLDL and decreased HDL cholesterol [160]. Cigarette smoke is a source of numerous oxidative compounds and heavy metals, which negative impact on PON1 has already been discussed in Paragraph 3.1.2. Not only unfavorable oxidative status impairs PON1 protective activity toward lipid peroxides, but also a large number of reactive compounds found in smoke, like aldehydes or aromatic hydrocarbons, has a direct inhibitory impact on PON1, which basically means the weakening of the natural defense mechanisms against ROS. These reactive chemicals directly interact with the PON1 active site, and the subsequent steric hindrance close to the region essential for substrate binding results in the enzyme’s inactivation [161]. Thiolation of lysine residues in PON1 structure is the other contributing mechanism [162].

Several studies independently confirmed a detrimental influence of smoking on PON1 activity [162,163,164,165,166]. Bizoń et al. [162], in addition to the negative relationship between cigarette smoking and PON1 activities, have investigated the impact of strong alcohol (40%) consumption, and their results confirm that it is negatively correlated with PON1 activity. Mouhamed et al. [160] extended their research to PON1 gene polymorphisms and found that smokers with 55MM genotype presented the lowest PON1 activity, while in 55LL individuals it was significantly higher. The investigation of polymorphism at position 192 led to the conclusion that the protective activity of PON1 was lower in the 192QQ allozyme bearers than in 192RR ones. Comparing the two SNPs, the change in position 55 compromised PON1 activity to a larger extent than the other one.

### 3.5. Pharmaceuticals

Drug molecules do not only affect their target proteins or receptors, but their activity can be addressed toward other macromolecules or enzymes, both of these participating in their metabolism and other. The list of pharmaceutical compounds investigated for their effects on PON1 tends to grow longer every year. In the following section, we will screen through some of the most frequently used groups of pharmaceuticals whose influence on PON1 has been assessed in the context of the character of the disease. It can be an important issue in the context of individuals with a high risk of CVD or atherosclerosis development, particularly since most drugs have been found to inhibit PON1 activity. A vast majority of studies (Table 1) was performed in vitro on purified human PON1 isolated from pooled serum. On one hand, this approach has many advantages as it eliminates some factors like age, gender, co-medications etc., but on the other hand does not provide the comprehensive view due to simplification of the conditions which, in vivo, are much more complex and correlated. The measurement of POase activity instead of AREase in most studies could be a limitation as well [167,168,169,170,171,172,173,174].

#### 3.5.1. Cardiovascular Drugs

Acetylsalicylic acid (ASA), known under the name aspirin, is undoubtedly one of most commonly used pharmaceuticals worldwide. The pharmacological effect of ASA is dose-dependent: low doses, typically 75 mg/day, are recommended for the prevention of cardiovascular events due to its antithrombotic activity, while higher doses have analgesic, antipyretic and anti-inflammatory effect [179]. Salicylates, the group of chemicals to which aspirin belongs, are administered in a form known at present for well over a hundred years, have been studied for their effect on PON1 since early 2000s and were shown to elevate both the concentration and activity of PON1. ASA intake was found to be associated with positive effects like increased ROS scavenging, encompassed lipoprotein oxidation and improved function of endothelium, which at least partially can be attributed to enhanced PON1 protective function [180]. These findings were confirmed in another study, where PON1 activity was induced by ASA, providing additional protection to prevent cardiovascular diseases and atherosclerosis [181]. Studies on cell cultures indicate that the effect of ASA on PON1 can originate from the induction of PON1 and apoA1 gene expression, probably mediated by AhR gene expression [182]. In addition, PON1 is involved in aspirin’s, and its derivative, nitro-aspirin’s metabolism [183].

The antihypertensive drugs tested, i.e., non-selective beta blockers (atenolol, nadolol, pindolol), midodrine—an α-1 adrenergic receptors agonist, and calcium channel blockers (nifedipine, nitrendipine, diltiazem and verapamil) were assessed to inhibit PON1 enzymatic activity in vitro with varying strength [176]. The inhibitory effect of digoxin, a cardiac glycoside acting via Na^+^/K^+^ ATPase activity suppression, on PON1 was most potent—according to the calculated IC_50_ value, it was able to inhibit PON1 in a micromolar range [177]. Such a strong effect implies that a significant reduction in biological free radical scavenging and detoxifying activity of PON1 might occur in vivo, and it would be particularly undesirable in patients suffering from cardiovascular diseases as it might predispose them to the disease progression on development other related health concerns.

#### 3.5.2. Pharmaceuticals Applied in the Treatment of Mood Disorders and Antiepileptic Drugs

It is now well documented that mood disorders (MDs) such as depression or bipolar disorder are connected with the increased risk of cardiovascular diseases in healthy subjects. This correlation can be an outcome of mixed physiological derangements observed in depression with behavioral mechanisms. In MDs, the imbalance between sympathetic and parasympathetic neurotransmitter modulation, increased cortisol levels, platelet activation and, in consequence, inflammatory response, mediated by cytokines (pro-inflammatory ILs, TNF-α, C-reactive protein), in the longer term lead to elevated blood pressure, arrhythmias and accelerates atherosclerotic lesions formation [184]. As PON1 activity is linked to diseases based on inflammatory and oxidative reactions, and MDs are considered as such, a few studies aimed to specify the relationship between PON1 activities and MDs. Most of their findings indicate that PON1 activities are lowered in individuals suffering from MDs [185].

Lithium, which has been applied in the treatment of bipolar disorder since the 1970s, is also an acknowledged PON1 inhibitor [186]. The next direction of research was to assess the influence of antidepressant drugs on PON1 activity. As the treatment is usually long-term, the supposedly already lowered PON1 activity might not provide sufficient protection, and increase the risk of cardiovascular incidents or disease development. Some classic tricyclic antidepressants, several pharmaceuticals representing a newer generation of selective serotonin reuptake inhibitors (SSRI) and antipsychotics have been investigated in the context of PON1 activity alterations. In general, tested compounds were proven to act as PON1 inhibitors in vitro [175]. Kati et al. [187], in their study on eleven SSRI-intoxicated patients, found significantly lower PON1 AREase activity and total antioxidant capacity with higher levels of oxidative stress markers in comparison to healthy controls. Contradictory information was obtained for fluoxetine, a major inhibitor of P_450_ system isozymes, which was determined by Avcikurt et al. [188] to inhibit PON1 activity in a noncompetitive manner, while Saadaoui et al. [189] had not observed any effect on PON1 within the therapeutic doses range. Remarkably, a pleiotropic substance, mirtazapine, which, apart from antagonizing serotoninergic and noradrenergic receptors, exerts cytoprotective effects and is involved in several antioxidant mechanisms’ activation, was found to have an inhibitory effect on PON1 in vitro. Therefore, these effects, in view of the reported study results, do not seem connected with the positive modulation of PON1 activity, and possibly some other pathways shall be involved.

According to Beydemir and Demir [171] in their in vitro study, antiepileptic drugs such as gabapentin or valproic acid have an inhibitory effect on PON1 activity. The impact of carbamazepine and valproic acid, which are still commonly prescribed, had been assessed in vivo in patients who begin their therapy, and PON1 activity was found to be significantly decreased in comparison to their pre-treatment levels. Additionally, a greater reduction of PON1 activity was observed for carbamazepine than for valproic acid in monotherapy [190]. What might be of relevance is that carbamazepine induces oxidative stress in cells [191].

#### 3.5.3. Antidiabetic Drugs

Several pharmaceuticals applied in T2D treatment were discovered to affect PON1 activity, including biguanide metformin and thiazolidinedione drugs represented by rosiglitazone. The latter have previously been known for its insulin resistance- and glucose-lowering effects in diabetic patients due to the enhancement of fatty acid uptake in adipose tissue with beta cell function and insulin sensitivity improvement. These features are attributed to the mechanism of action—rosiglitazone is an agonist for nuclear PPAR-γ receptors which control and regulate genes involved in lipid and carbohydrate metabolism, determining the response to lipid and glucose intake [192]. It was found to decrease free radicals and exert a positive effect on the lipid profile manifested by elevated serum HDL levels, increased HDL-apoA1 synthesis and changes in HDL size distribution toward the synthesis of smaller HDL subclasses, which in turn may influence PON1 activity [193]. Following what has already been mentioned about PON1 expression being upregulated via PPAR-mediated pathway, it was supposed that the antioxidant and anti-inflammatory effects observed after rosiglitazone treatment can be partly associated with the positive regulation of PON1 activity. These assumptions have been confirmed with studies in animal models and small human cohorts. Atamer et al. [194] observed, in diabetic patients treated with rosiglitazone, a significant increase in serum apoA1 and HDL cholesterol levels along with POase PON1 activity. Another study, placebo-controlled and double-blind, had shown that T2D patients assigned to rosiglitazone exhibited increased fasting PON1 activity and significantly reduced plasma peroxide levels [195].

There are some reports available concerning the influence of metformin on PON1 activity, which is regarded as the most commonly used T2D antidiabetic. A 2012 randomized clinical trial investigating effects of metformin on oxidative stress markers revealed a significant increase in PON1 activity in a studied population with a reduction of advanced oxidation protein products (AOPPs) and advanced glycation end products (AGEs)—oxidative stress markers [196]. Similar results of PON1 and catalase activity increase with a concurrent significant reduction in AOPP and lipid peroxides levels were obtained in a 2020 study on 40 diabetic patients [197]. Data gathered by Meaney et al. [198], who observed a raise in PON1 activity in metformin-treated patients with metabolic syndrome with no significant increase HDL levels, suggest that there is a correlation between PON1 activity and carotidal intima-media thickness reduction. These findings concerning positive impact of metformin on PON1 activity are consistent to observations gathered in animal studies [199]. Based on the current state of research, it is suspected that the mechanism of metformin’s therapeutic effect could include the upregulation of PON1.

#### 3.5.4. Drugs Regulating Lipid Metabolism

PON1 activity is known to be reduced in patients suffering from hypercholesterolemia and obesity, and it was established as a predictive factor of cardiovascular diseases [200,201]. an inverse correlation between PON1 AREase activity and BMI is thought to result from elevated oxidative stress, which accompanies metabolic disorders such as obesity [202]. In obese individuals, intensified processes of LDL and HDL oxidation are connected with the secretion of numerous cytokines by adipose tissue, among which leptin has a crucial role. Its increased expression, proportional to adipose tissue content, has a pro-inflammatory and pro-atherosclerotic effect, mainly via induction of ROS generation. The findings of Bełtowski et al. [203] suggest that leptin is a major factor responsible for decreased PON1 activity observed in obesity. Leptin was shown to decrease plasma HDL and triglyceride levels and is supposed to interact with apoA1; therefore, the alterations in HDL subfractions modulate PON1 activity and influence cellular metabolism of lipids.

The use of orlistat, whose mechanism of action relies on gastrointestinal lipase inhibition resulting in a decrease of fat absorption, is one of the most promising opportunities in the pharmacological control of obesity. Independently, orlistat was shown to reduce cholesterol levels [204], and PON1 activity was demonstrated to significantly increase after a 6-month-long treatment with orlistat in comparison to pre-treatment status; however, the precise mechanism needs to be further investigated. This beneficial effect can be due to oxidative stress reduction and lipid redistribution between HDL subfractions increasing the ratio of the small dense HDL3 subclass. Certainly, the antioxidant status improvement is a valuable aspect in orlistat intake [205].

3-hydroxy-3-methylglutaryl-CoA reductase inhibitors, known as statins, are often the first choice of pharmaceuticals in increased levels of serum cholesterol. Numerous studies in vitro and on cell cultures aiming to explain implication of statins in serum PON1 activity have been conducted, but the results are conflicting. The majority of studies on human cell lines have shown a positive modulation of PON1 activity in response to atorvastatin, and a similar effect was reported for simvastatin and pivastatin [206,207], while one study on human hepatoma cells reported a decrease in PON1 activity [208]. The effect of statins exerted on PON1 seems to be connected with the activation of transcription factors Sp1 and SREBP-2 through the MAPK-mediated pathway, which upregulates PON1 promoter activity and subsequent gene expression [209,210]. Yet, the positive impact of statin therapy on PON1 activity was only evidenced in small studies on dyslipidemic patients [206,211] and a group of patients suffering from primary hyperlipoproteinemia [212]. Interestingly, Mirdamadi et al. [213] connected the efficacy of atorvastatin treatment with a phenotype determined by PON1 Q192R polymorphism.

Ezetimibe, a selective inhibitor of cholesterol and phytosterol absorption in the small intestine, was found to be one of the few pharmaceuticals that stimulate PON1 activity. A study on thirty hyperlipidemic patients treated with ezetimibe (a selective cholesterol absorption inhibitor) has demonstrated an increase in PON1 activity with a general improvement in total oxidative status [214]. Patients with metabolic syndrome included to Naoku et al. [215] randomized study, who have been treated with ezetimibe and orlistat or these two drugs combined, showed significant increase in PON1 activity and PON1/HDL-C ratio.

#### 3.5.5. Oral Contraceptives

Today, oral contraceptives (OCs) is the most commonly used contraception method. OC intake was formerly linked to oxidative stress induction, manifested by an increased level of γ-glutamyltransferase, and with alterations in lipid profile due to interactions with estrogen receptor [216], with the ultimate effect depending on the exact composition of the pharmaceutical. According to the oxidative stress conditions and hepatic apolipoprotein upregulation attributable to OC intake, Kowalska et al. [217] recognized a likely connection between these and altered PON1 activity. Indeed, they have demonstrated an increase in PON1 AREase and LACase activity. The latter might indicate that PON1 is involved in OC metabolism, while higher AREase activity can be a response to the exposure of increased ROS formation. A decrease in the phosphotriesterase activity of PON1 was observed as well, and it can be stated that the influence of OCs themselves and accompanying alterations in a cellular microenvironment on PON1 regulation is complex. Given that these drugs are metabolized by the liver, authors speculate that PON1 phosphotriesterase activity might be an indicator of liver condition along with the activity of alanine aminotransferase (ALT), aspartate aminotransferase (AST) and γ-glutamyltranspherase (GGT) in women taking OCs. These results are consistent with findings of a cohort study conducted by Vincent-Viry et al. [218], who demonstrated higher PON1 levels along with its POase and AREase activity in women taking OCs compared to the group which was not treated with OCs, and with results of the research of van den Berg et al. [219].

#### 3.5.6. Pharmaceuticals Used in Chemotherapy

There are limited data concerning human antioxidant response to antineoplastic drugs and chemotherapy in general. Several pharmaceuticals tested in vitro have been shown to inhibit PON1 in either a competitive or noncompetitive manner (Table 1). The IC_50_ values calculated for paclitaxel, cetuximab and bevacizumab are expressed in the micromolar range. The plant-derived group of quinone compounds, although acknowledged to be promising in terms of potential future antitumor therapies targeted toward free radicals generation, had been found to inhibit PON1 activity as well [220].

Recently, PON1 AREase and POase activity has been assayed in a cohort of women with breast cancer treated with doxorubicin (DOX) to determine whether there is any association with cancer therapy-related cardiac dysfunction development (CTRCD) [221]. DOX-induced cardiotoxicity is largely a result of oxidative stress, which, apart from the direct damage of cardiomyocytes through mitochondrial dysfunction and apoptosis, induces metabolic dysfunction of endothelial cells, thereby compromising endothelium barrier function, disrupting paracrine signaling to cardiomyocytes and decreasing nitric oxide release [222]. A decrease in both determined in this study PON1 activities was observed with time as a result of exposure to DOX, which indicates the utilization of enzyme molecules in a response to accumulating toxin. This effect may also be related to limited PON1 synthesis in the liver as DOX negatively affects the hepatic synthetic function. Remarkably, it was found that an early increase in POase activity after DOX completion is correlated with an increased risk of CTRCD in this group of patients, which suggests the potential clinical relevance of PON1 activity measurement. It has been previously shown that an increase in PON1 activity can be related to a rise in the ApoA1 catabolic rate, which is associated with the HDL switch to a subtype characterized by decreased cardioprotective capacity [221].

## 4. Non-Modifiable Factors Modulating PON1 Activity

### 4.1. Genetic Polymorphisms

Gene promoter polymorphisms are assessed to account for about 25% of variation in PON1 serum levels. It was therefore extensively studied how genetic variability affects the catalytic activity of PON1. As for 2018, eight SNPs in the promoter and over 200 in the coding region have been identified, of which two seem to be clinically relevant—the substitution of leucine (L) to methionine (M) at position 55 and a glutamine (Q) to arginine (R) at codon 192 [223,224]. Q192R is the most widely studied PON1 polymorphism due to significant variation in the catalytic efficacy of the two allozymes and substrate affinities, which majorly affects activity toward OPs. Individuals with an R allele have been found to hydrolyze paraoxon about six times faster than those possessing Q in this position and hydrolyze HCTL more efficiently [20]. On the other hand, diazoxon, sarin and soman are more rapidly hydrolyzed by PON1 192Q allozyme. Such a significant alteration caused by SNP might be connected with the location of R residue within the enzyme’s active site [223]. The polymorphism at position 192 is considered a determinant indicator of oxidant status, as Q192 allozyme demonstrates a higher efficacy against oxidized lipids and LDL oxidation than the R form. However, hydrolytic rates for phenylacetate and dihydrocoumarin have not been demonstrated to differ between polymorphic variants, which indicates a dependency on the substrate type [225]. AREase activity is therefore regarded to be genotype-independent and is preferably determined in studies conducted in vitro.

The L55M polymorphism has been determined to not affect PON1 activity, but instead serum PON1 levels where M55 individuals are characterized by lower PON1 concentrations. About 98% of the R192 allele carriers were determined to have L at position 55; thus, it can be referred to as a strong disequilibrium between these alleles [226]. The L55 isoform is regarded to have a higher stability and resistance to proteolysis [227]. In addition, as is evident from the PON1 crystal structure, L in position 55 is a crucial component determining protein structure to be packed properly [10]. In numerous studies, it has been attempted to address the relation between L55M polymorphism and cardiovascular or cerebrovascular diseases. Recent research suggests that the 55M allele is associated with glycemic control in T2D patients. It was observed that glycemic control improves in order of genotype from LL through LM to MM [228], suggesting improved pancreatic β-cell function related to the M allele. These findings support Chiu et al.’s [229] assumption that L55M plays a role in β-cell function and variation in insulin response and Barbieri et al.’s [230] conclusion that the LL genotype can be considered a predictor of insulin resistance. Moreover, in a 2013 meta-analysis [231], patients with an L55 allele have been shown to present a higher risk of diabetic nephropathy occurrence. Recent evidence positively correlates the allelic frequency of the M55 allele with the higher prevalence and mortality of COVID-19 [232].

**Figure 3:**
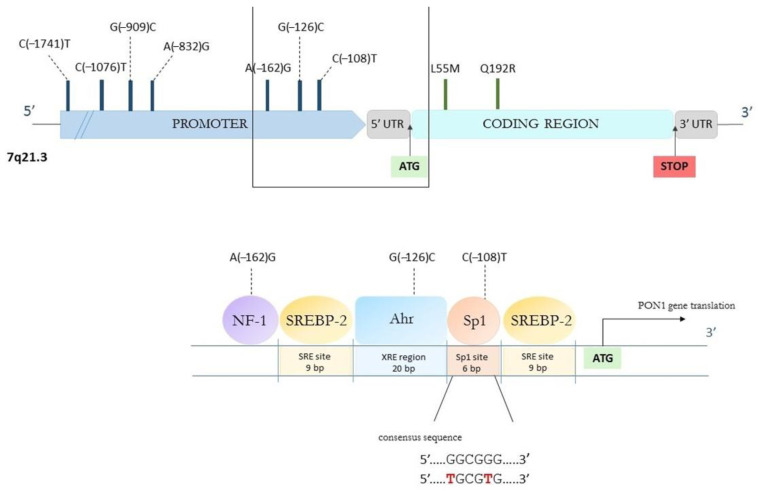
Polymorphic variants on PON1 gene promoter and coding region.

The sequencing of the PON1 gene revealed several SNPs in both 5′ and 3′ untranslated region (UTR) and eight polymorphisms in the promoter (non-coding) region at present, among which, cytosine (C) to thymidine (T) substitution at position −108 seems to be major. It accounts for about 23% of the PON1 serum expression variation, where C is the high-activity variant [226]. The importance of C(−108)T polymorphism in affecting PON1 serum levels can be explained with its location within the consensus sequence (5′…GGCGGG…3′) in the binding site of transcription factors Sp1 and Sp3 (specificity protein 3). The presence of T as a −108 variant interferes with this consensus sequence and seems to weaken Sp1 binding to −108 site, lowering PON1 gene expression and PON1 synthesis [227]. In comparison, the contribution of A(−162)G polymorphism in the total PON1 variation is approximately 1%. It is suspected to relate to the location of this polymorphism within the potential nuclear factor-1 (NF-1) binding site for A variant connected to high PON1 activity. NF-1 is known to be a ubiquitous transcription activator; therefore, −162A alleles upregulate gene expression while G variant does not form the binding site properly [233,234]. This has been presented in Figure 3. Another noteworthy PON1 polymorphism is an A to G substitution located in exon 4, which results in an isoleucine to valine substitution at codon 102 (I102V). A study on a large cohort of Finnish men revealed a correlation between the occurrence of the V allele and an increased risk of prostate cancer, as carriers of this mutation have been determined to present a higher relative risk for developing this disease during follow-up [235].

Due to its role in the modulation of CVD risk through the antioxidant and antiatherogenic activities of the PON1 enzyme, the *PON1* has been proposed as a candidate for the human longevity gene since the oxidation of lipids and the following changes in metabolism are major contributors to the development and progression of chronic diseases in the elderly. However, works aiming to answer the question whether specific PON1 polymorphisms affect longevity that have been published so far give contradictory results. A meta-analysis of 11 previously reported studies conducted by Lescai et al. [236], which included almost 6000 individuals, demonstrated that the PON1 variant at codon 192 does influence the likelihood of reaching extreme ages, and this probability is significantly higher in subjects possessing the RR or QR genotype. Nevertheless, another later published meta-analysis [237] indicated that the effect of *PON1* polymorphisms on human longevity, if it occurs, might only be population-specific and cannot be extrapolated for the general population.

### 4.2. Age

It has been identified that PON1 activity values at the time of human fetal development are low and start to increase between 6 and 15 month after birth. Hence, children under 15 months of age are more susceptible to the toxic effects of OPs. In addition, these compounds penetrate placenta and breast milk and are able to cross infants’ blood–brain barrier, which is still in development until it is completely formed at about 1 year old [238]. It is believed that after 15 month of age, PON1 activity reaches the plateau phase and remains at a similar level until elderly age. However, more recently, Huen et al. [239] reported that serum levels of PON1 may rise until 5 years of age.

The processes of aging are closely connected with increased oxidative stress [240]. The oxidative status in the elderly population is characterized by insufficient antioxidant synthesis, impaired absorption of antioxidant compounds from food and weakened enzymatic activity of catalase and superoxide dismutase [241]. Additionally, the activity of protein tyrosine phosphatases—proteases decomposing proteins modified under oxidative stress conditions, which trigger improper cellular signaling pathways altering numerous physiological processes and enzymatic activities—is reduced [242]. Elevated concentrations of acute phase reactants induce inflammatory conditions in cells. It altogether leads to the peroxidation of fatty acids and lipids, decreasing the cellular ability to regenerate and promoting their senescence, and contributes to a higher prevalence of certain diseases related to lipid oxidation and inflammation [243].

An age-related decrease in the antioxidant capacity of HDL is also a known fact [244]. It was therefore posed that the reduction in the antioxidant potential and antiatherogenic properties of HDL might be associated with lowered PON1 activity. Jaouad et al. [245] compared the antioxidant activity for young and elderly individuals and concluded that the observed decrease in the latter group is related to reduced PON1 activity. They later identified that it is due to alterations in PON1 free sulfhydryl groups and the following reduction of functional -SH content. Complementarily, increased free radicals formation turns HDL susceptible to oxidation with age and affects its physico–chemical properties, which is reflected in PON1 reduced activity. In turn, decreased PON1 activity considerably increases HDL proneness to oxidation [243]—PON1 and HDL act as a complex; therefore, its total functionality depends on both constituents. Other than that, HDL from the small healthy elderly group [246] showed lower POase PON1 activity with truncation and multimerization of apo-A1, and increased triglyceride and AGEs content which, overall translate to lower antioxidant ability.

Milochevitch and Khalil [247] reported that PON1 activity significantly decreased in the elderly group in comparison to the young subjects with no relation to PON1 phenotype. Similarly, Seres et al. [248] observed an age-dependent loss of PON1 AREase and POase activity with no concurrent change in either PON1 or HDL serum concentration. The authors point at the role of increased total cholesterol, which is also connected with aging, in the development of atherogenic conditions leading to partial PON1 inactivation and speculate that an observed age-dependent decrease in apo-A1 content and lipid redistribution among subclasses of HDL may also contribute. Another report by Mehdi and Rizvi [249] based on studies on the Indian population confirmed that PON1 activity decreases with age. The reduction in PON1 AREase activity has been proven to correlate with the proneness of LDL to oxidation and is connected to free radical scavenging activity observed in plasma. It is consistent with the knowledge of PON1 being inhibited by accumulated oxLDL. Consequently, PON1 is not able to sufficiently prevent lipoproteins from oxidation and remove already generated oxidized lipids. Loued et al. [250], by incubating oxLDL with HDL and PON1 purified from the plasma of the young and, separately, from elderly subjects, discovered that the PON1 of young persons presented the highest activity against the expression of ICAM-1. Other than that, the PON1 obtained from young subjects’ plasma was effective in monocyte chemotaxis inhibition, while elderly PON1 did not exhibit significant activity aimed at their chemotactic activity. One study suggested that polymorphic variants of PON1 in position 192 determine the extent of PON1 activity reduction—in QQ homozygotes, a greater loss in PON1 activity with age was assessed [251].

### 4.3. Gender

The third non-modifiable factor influencing serum PON1 activity is gender. Like it was observed in animal models, epidemiological studies in humans on over 1400 subjects confirmed that PON1 activity has significantly higher values in women than in men [252]. The study based on female hormone supplementation in a group of postmenopausal women, however, did not conclusively clarify this issue. According to results provided by Fenkci et al. [253], intranasal administration of estradiol did not affect PON1 activity, whereas hormone replacement therapy with the use of estrogen and progesterone was observed to increase it [254]. Notably, the group of women who were subjected to hormone replacement therapy suffered from diabetes, which might influence the conditions. Taken together, it is suspected that cell-associated PON1 activity is enhanced by estradiol, which also regulates surface PON1 stability but does not affect PON1 mRNA expression [255], and/or the male-pattern growth hormone might downregulate PON1 expression resulting in its low activity in men [256].

## 5. Conclusions and Future Perspectives

Recent decades brought great progress in the field of HDL metabolism and functionalities, along with its unique lipoprotein-bound enzyme, which is PON1. The impressive research initially allowed us to determine that PON1 accounts for antioxidant and atheroprotective properties of HDL; this discovery was followed by the constant gathering of information on its interaction with other factors involved in cellular protection and metabolic processes and the exploring of PON1 expression pathways. It enabled the addition of another piece to the knowledge concerning human homeostasis and regulatory processes. We have underlined the importance of epigenetic factors regulating PON1 activity, such as the composition of dietary lipids and sugars, elements of lifestyle such as alcohol intake and tobacco smoking, heavy metal exposure or medications taken, and described modifiable and non-modifiable variables which are able to interfere with PON1 and alter some of its activities toward different substrates, based on the most recent reports available.

In light of the results of studies reviewed in this manuscript, it is evident that PON1 activity is observed to decrease with age and generally takes higher values in women than in men. In addition, genetic polymorphisms are a PON1-modulating factor that shall not be neglected, as their further study can provide knowledge concerning, e.g., the efficacy in metabolizing pharmaceuticals or individual antioxidative capacity. It can also be concluded that a diet rich in polyphenols, monounsaturated fatty acids and characterized by moderate alcohol—in particular, dry red wine consumption—is beneficial in terms of PON1 activity and, therefore, its antioxidant capacity. The opposite effect is related to a high intake of oxidized lipids present in saturated fatty acids and glucose. Tobacco smoking and consuming high amounts of alcohol undoubtedly decreases both PON1 concentration and activity. It has to be kept in mind that most of pharmaceuticals from different groups that are often frequently used, such as several antihypertensive drugs, some antibiotics and drugs applied in mood disorders treatment, as assessed in numerous in vitro studies, are also able to inhibit PON1 activity.

From our review, it is visible that PON1 could be a great target for intervention due to its multifaceted functions and involvement in a number of physiological processes. A better understanding of factors which alter PON1 functionality, both positively and negatively, can contribute to the future development of PON1 activation mechanisms which could be valuable as new methods of control and treatment of common 21st century diseases and disorders. Further investigation regarding mechanisms and factors involved in PON1 activity modification can be of clinical importance in atherosclerotic disease management in the future. Moreover, the fact that PON1 activity and alterations of lipoprotein metabolism present a similar pattern both in atherosclerosis and cancer deserves further exploration in the search for a correlation between CVD and the development of cancers. If the hypothesis about a crossroad between these two diseases could be confirmed, new strategies to prevent or treat them simultaneously could be introduced to clinical practice. Additionally, there is a knowledge gap regarding PON1 regulatory pathways in vivo, involving miRNAs or epigenetic factors contributing to its activity modulation, which would be essential to fill in order to enable the discovery of new PON1 effectors for therapeutic applications.

## Figures and Tables

**Table 1:** List of PON1-inhibiting drugs grouped according to their application. The half maximal inhibitory concentration (IC_50_) and inhibition constant (K_i_) values have been calculated by authors based on paraoxonase activity measurement. Types of inhibition include competitive (C), uncompetitive (UC) or noncompetitive (NC).

Class	Drug	Type	IC_50_ (mM)	K_i_ (mM)	Reference
Antihypertensives	atenolol	NC	0.136 ± 0.003	0.092 ± 0.013	[167]
	nadolol	C	0.214 ± 0.008	0.075 ± 0.016	
	pindolol	NC	0.370 ± 0.008	0.287 ± 0.028	
	midodrine	C	0.131 ± 0.007	0.056 ± 0.007	
Antimycotics	amphotericin B	NC	0.266 ± 0.002	0.320 ± 0.020	[168]
	fluconazole	C	5.728 ± 0.043	2.546 ± 0.166	
	caspofungin	C	0.037 ± 0.001	0.011 ± 0.002	
Proton pump inhibitors	pantoprazole	C	54.78 ± 0.524	39.90 ± 0.005	[169]
	omeprazole	C	86.47 ± 0.818	70.11 ± 0.010	
	esomeprazole	C	93.39 ± 0.885	78.87 ± 0.008	
Sedatives	midazolam	NC	0.085	0.057 ± 0.006	[170]
	diazepam	NC	0.104	0.181 ± 0.019	
Antiepileptics	gabapentin	NC	0.350	0.261 ± 0.027	[171]
	phenytoin	NC	6.300	10.30 ± 0.001	
	valproic acid	NC	0.670	0.338 ± 0.313	
	primidone	NC	0.870	0.410 ± 0.184	
	levetiracetam	NC	53.30	43.01 ± 0.003	
Anesthetics	etomidate	NC	0.021	0.059 ± 0.014	[172]
	propofol	C	0.328	0.322 ± 0.111	
	ketamine	UC	3.800	6.480 ± 0.963	
Antineoplastics	cetuximab	NC	0.011	0.019 ± 0.004	[173]
	paclitaxel	C	0.042	0.017 ± 0.011	
	docetaxel	NC	0.665	0.291 ± 0.108	
	etoposide	NC	0.226	0.131 ± 0.071	
Antibiotics	cefuroxime	C	13.78	6.642 ± 1.541	[174]
	cetriaxone	UC	13.37	6.427 ± 0.912	
	ceftazidime	NC	15.06	12.79 ± 1.124	
	teicoplanin	NC	0.077	0.090 ± 0.007	
	rifamycin	C	0.306	0.716 ± 0.076	
	tobramycin	UC	5.170	3.381 ± 0.406	
	amikacin	NC	40.76	55.44 ± 7.783	
Selective serotonin reuptake inhibitors	mirtazapine	NC	0.231	0.276 ± 0.035	[175]
	aripiprazole	NC	0.139	0.202 ± 0.031	
	escitalopram	C	0.173	0.047 ± 0.004	
	risperidone	C	0.116	0.042 ± 0.004	
Calcium channel blockers	nifedipine	C	0.121	0.222 ± 0.049	[176]
	nitrendipine	C	0.130	0.151 ± 0.067	
	israpidine	C	0.255	0.286 ± 0.137	
	amlodipine	NC	0.304	0.321 ± 0.002	
Cardiovascular drugs	verapamil	UC	0.672 *	1.188 ± 0.115 *	[177]
	metoprolol	NC	0.621	1.115 ± 0.270	
	digoxin	NC	0.012	0.035 ± 0.0127	
	diltiazem	NC	1.462	3.104 ± 1.005	
	amiodarone	C	3.255	5.427 ± 1.341	
	dobutamine	UC	4.495	10.7 ± 3.146	
Ophthalmic drugs	travoprost	C	14.95 *	9.71 ± 2.63 *	[178]
	latanoprost	NC	17.03 *	31.69 ± 3.38 *	
	olopatadine	C	299.6 *	261.5 ± 59.98 *	
Other	ketotifen	NC	87.29 *	239.9 ± 33.25 *	[178]
	methylprednisolone	C	47.80	109 ± 17.479	[177]

* values expressed in μM.

## Data Availability

Not applicable.
